# Causal Relationship between Aging and Anorexia Nervosa: A White-Matter-Microstructure-Mediated Mendelian Randomization Analysis

**DOI:** 10.3390/biomedicines12081874

**Published:** 2024-08-16

**Authors:** Haoyuan Qiu, Miao Shi, Zicheng Zhong, Haoran Hu, Hunini Sang, Meijuan Zhou, Zhijun Feng

**Affiliations:** 1 School of Biomedical Engineering, Southern Medical University, Guangzhou 510515, China; nickxqq777@foxmail.com (H.Q.); esthermiaoshi@outlook.com (M.S.); lucas.zsling@gmail.com (Z.Z.); h1275477946@gmail.com (H.H.); 2 School of Pharmaceutical Sciences, Southern Medical University, Guangzhou 510515, China; sowelive0825@gmail.com; 3 Department of Radiation Medicine, Guangdong Provincial Key Laboratory of Tropical Disease Research, School of Public Health, Southern Medical University, Guangzhou 510515, China

**Keywords:** anorexia nervosa, aging, telomere length, white matter, magnetic resonance imaging, Mendelian randomization

## Abstract

This study employed a two-step Mendelian randomization analysis to explore the causal relationship between telomere length, as a marker of aging, and anorexia nervosa and to evaluate the mediating role of changes in the white matter microstructure across different brain regions. We selected genetic variants associated with 675 diffusion magnetic resonance imaging phenotypes representing changes in brain white matter. F-statistics confirmed the validity of the instruments, ensuring robust causal inference. Sensitivity analyses, including heterogeneity tests, horizontal pleiotropy tests, and leave-one-out tests, validated the results. The results show that telomere length is significantly negatively correlated with anorexia nervosa in a unidirectional manner (*p* = 0.017). Additionally, changes in specific white matter structures, such as the internal capsule, corona radiata, posterior thalamic radiation, left cingulate gyrus, left longitudinal fasciculus, and left forceps minor (*p* < 0.05), were identified as mediators. These findings enhance our understanding of the neural mechanisms, underlying the exacerbation of anorexia nervosa with aging; emphasize the role of brain functional networks in disease progression; and provide potential biological targets for future therapeutic interventions.

## 1. Introduction

Anorexia nervosa (AN), a severe psychiatric disorder with a lifetime prevalence of 0.80% to 3.60%, is primarily characterized by malnutrition and starvation [1]. The global incidence of AN is increasing, particularly in Asia and the Middle East [2,3]. As the psychiatric disorder with the highest mortality rate, AN often coexists with other psychiatric conditions, such as major depression, anxiety disorders, and trauma-related disorders. It exhibits resistance to treatment and carries a significant risk of death due to medical complications and suicide [4,5,6]. The long-term prognosis of AN varies: 30 to 60% of patients achieve full remission, 20% experience chronic illness, and the remainder exhibit residual symptoms [1]. A recent genome-wide association study identified eight risk loci for AN, which are also associated with other psychiatric disorders, low BMI, and metabolic abnormalities [7]. Research indicates that the development of AN is influenced by a combination of sociopsychological factors and genetic background. Family and twin studies have consistently demonstrated a significant genetic component to AN, with heritability estimates ranging from 50% to 60%. Recent genome-wide association studies (GWAS) have identified several genetic variants associated with AN. These variants are implicated in multiple biological pathways, including neurotransmitter systems, energy metabolism, and immune function. These findings provide valuable insights into the biological underpinnings of AN and have the potential to inform the development of more effective treatments [8,9]. Telomeres are repetitive sequences located at the ends of each human chromosome. Their primary function is to prevent the inappropriate activation of DNA damage repair mechanisms, which could lead to telomere fusion and genomic instability [10]. With each round of DNA replication and subsequent cell division, telomere length (TL) progressively shortens [11]. This incremental shortening eventually triggers cells to enter an irreversible state known as replicative senescence, contributing to the process of aging in humans [12,13,14,15]. Abnormally short or dysfunctional telomeres can result in a group of rare, heterogeneous diseases characterized by premature aging, such as dyskeratosis congenita, pulmonary fibrosis, osteoporosis, and various psychiatric disorders [10]. Additionally, Cawthon et al. found that in an elderly population aged 60 years and older, telomere length in the blood was significantly associated with the risk of death. Their study revealed that for every standard deviation reduction in telomere length, the risk of death increased by 25% [16].

Recent findings have identified TL as a biomarker associated with regional brain size, independent of age [17]. Research by Mikael Wikgren and colleagues provides evidence of an association between TL and cerebral subcortical atrophy and white matter hyperintensities, supporting the concept of telomere length as a marker of biological aging, particularly in relation to the aging brain [18]. The incidence of psychiatric disorders, including AN, significantly increases in the elderly population; however, the specific mechanisms remain unclear. With advancing age, brain volume gradually decreases, and structural abnormalities in certain brain regions may be major contributors to the development of these disorders. Nevertheless, the causal relationships involved are not yet well-defined [17,18]. Future research is needed to determine the association between regional brain structural changes and psychiatric disorders like AN.

The white matter of the brain is composed of nerve fibers wrapped in myelin sheaths, which are primarily made of lipids. Its main function is to speed up the transmission of nerve impulses and improve the efficiency of neural communication. The brain’s white matter is critical for cognitive function and is involved in sensory processing, motor control, and more complex functions, such as learning and emotional regulation. Studies have shown that abnormalities in the brain’s white matter are strongly associated with a variety of psychiatric disorders, such as schizophrenia and bipolar disorder. Patients with these conditions often exhibit structural and functional impairments in white matter pathways. These impairments can lead to decreased efficiency in nerve signaling, consequently affecting the patient’s cognitive and emotional responses. Therefore, a deeper understanding of the physiological function of the brain’s white matter and its role in psychiatric disorders is of great significance for developing new treatments [19,20].

Diffusion magnetic resonance imaging (dMRI) is a widely used method by neuroscientists to obtain unique information about structural connections in the brain [21]. As a non-invasive imaging technique, dMRI examines white matter pathways by quantitatively measuring the distribution and movement of water molecules in tissues, mapping anatomical connections using fiber-tracing techniques [21,22,23]. dMRI technology relies on various diffusion metrics, with changes in these metrics revealing alterations in the white matter microstructure. Advancements in dMRI technology have led to the development of more complex tensor models and computational methods, such as Tract-Based Spatial Statistics (TBSS) and the ProbtrackX toolbox (PXTB), to better describe the intricate structure of white matter. The TBSS method enhances the accuracy of non-linear image registration by constructing a standard skeleton of fiber tracts based on fractional anisotropy (FA) images from all subjects. This FA skeleton is used to represent the fiber tracts, a technique not utilized by the PXTB [21,24,25]. Research indicates that AN is likely related to changes in the brain’s white matter microstructure, suggesting that certain dMRI phenotypes may be associated with this disorder [26].

Mendelian randomization (MR) analysis, an emerging method of causal inference, uses genetic variation as an instrumental variable (IV) to effectively avoid confounding bias and reverse causation issues commonly encountered in traditional observational studies [27]. Recent studies have demonstrated the wide-ranging applications of MR in the field of health. For instance, Larsson et al. explored the associations between genetic predispositions to smoking behavior and multiple cardiovascular diseases, revealing the long-term health impacts of smoking [28]. Additionally, Khera et al. utilized MR to predict the genetic influence on weight and obesity trajectories, offering new insights into the risk of adult obesity [29]. These MR analysis studies deepen our understanding of the role of genetic factors in disease development and provide a theoretical basis for personalized medical strategies.

This study aims to systematically evaluate the impact of TL on AN and the mediating role of the white matter microstructure in AN through MR analysis. This approach will elucidate the causal relationships between TL, white matter microstructural degradation, and AN. The objectives of this study are to explore the one-way causal relationship between TL and the severity of AN, assess the mediating role of white matter microstructural degradation in the relationship between TL and AN, and provide insights into potential targets for AN prevention and treatment based on findings related to TL and white matter microstructural degradation. Through this study, we hypothesize that changes in TL may increase the risk of AN by affecting the integrity of white matter microstructures in various brain regions. Additionally, we hypothesize that AN does not influence changes in TL in any manner. This study aims to provide new perspectives on the etiology of AN and offer a scientific basis for future prevention and treatment strategies.

## 2. Materials and Methods

### 2.1. Study Design and Data Source

This study employed a bidirectional and two-step approach to investigate the potential causal relationship between TL and AN and to assess the mediating effect of white matter phenotypes in this causal pathway. The first step involved MR analysis based on genetic variants to explore the causal relationships between TL and AN, TL and white matter phenotypes, and white matter phenotypes and AN. The second step evaluated the mediating effect of white matter phenotypes in these relationships and quantified their proportion using the coefficient product method, thus revealing the potential significance of white matter phenotypes in the influence of TL on AN.

Following the STROBE-MR guidelines, we adopted a bidirectional two-sample MR approach [30]. Summary statistics for TL were obtained from the UK Biobank, which included up to 472,174 participants of European ancestry [31]. Summary statistics for AN were sourced from the Psychiatric Genomics Consortium GWAS, encompassing a cohort of 72,517 individuals of European descent, segmented into 16,992 cases and 55,525 controls [32]. For external validation, we used AN GWAS data from the FinnGen database (452 cases, 411,729 controls) [33].

Considering that dMRI can better reveal changes in the brain’s white matter microstructure, we extracted information on 675 dMRI phenotypes from 3144 intrinsic brain activity phenotypes in the UK Biobank (n = 8428). These phenotypes were processed using TBSS and the PXTB [25]. Our analysis focused on these 675 dMRI phenotypes to explore the mediating effects of white matter function and structure. All detailed descriptions of the phenotypes of dMRI parameters are presented in Table A1. In cases of missing information in the summary statistics, such as effect allele frequency, we utilized the matched human genome build as a reference to complete the data (Table S1). The datasets used were from large case samples of the same ethnicity but from different regions, which helps to mitigate the bias risk from sample overlap in MR analysis.

**Figure 1:**
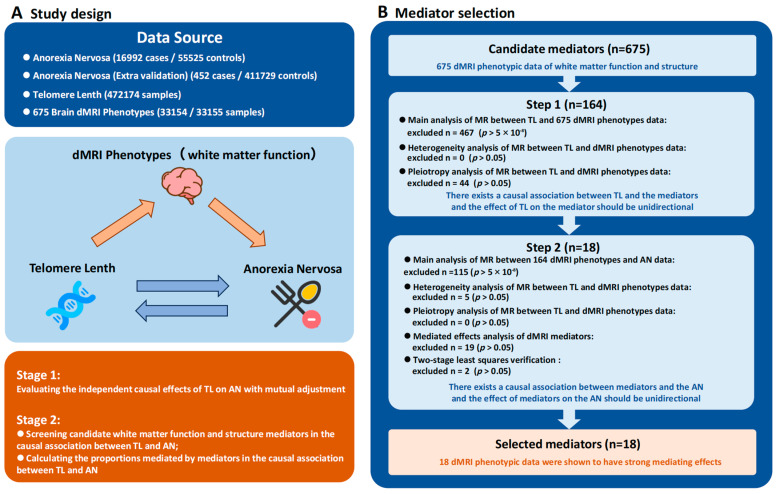
Study design and mediator selection method.

In the forward MR analysis, TL was set as the exposure factor, with AN and white matter phenotypes as the outcomes. Genetic variants strongly associated with the exposure factor were selected as instrumental variables (IVs). These IVs must satisfy three key conditions to ensure the validity of the MR analysis [27]: (1) They must be strongly associated with the exposure factor; (2) they must be independent of potential confounding factors; and (3) their effect on the outcome must operate exclusively through the exposure factor and not through other pathways. As the data used in this study were derived from publicly available GWAS summary statistics, no additional ethical approval or informed consent was required. The specific process of experimental design can be seen in Figure 1.

### 2.2. IVs Selection

In the MR analyses, IVs identified through GWAS are utilized as proxies to elucidate the gene-level causal relationships between genetic exposures and outcomes. For this purpose, only IVs that achieved a genome-wide significance threshold (*p* < 5 × 10^−8^) were retained. We excluded IVs in linkage disequilibrium (LD) outside of a 10,000 kb window and with an r^2^ < 0.001. Additionally, we excluded IVs that exhibited a significant association with the outcomes (*p* < 5 × 10^−8^).

To limit the influence of confounding factors, we further removed IVs associated with the outcome using the LDlink database (accessed on 26 April 2024) and the GWAS catalog (https://www.ebi.ac.uk/gwas/, accessed on 27 April 2024) [34,35]. The F-statistic for each IV was calculated using Formula (1) to assess instrument strength. IVs with an F-statistic greater than 10 were selected to mitigate the risk of weak instrument bias [36].

(1) $$F=\frac{R^{2}\left(N-K-1\right)}{K\left(1-R^{2}\right)}$$

To examine potential influence from reverse causality, the ‘Steiger test’ method was applied using the ‘TwoSampleMR’ R package [37]. This test scrutinizes MR associations that passed the multiple-testing threshold. The results were categorized as ‘true’ if the effect direction was from exposure to outcome with *p* < 0.05, as ‘false’ if reversed with *p* < 0.05, and as ‘uncertain’ if *p* ≥ 0.05. This method helps confirm the validity of the causal direction inferred from the MR analysis [38].

### 2.3. MR Analysis

A bidirectional and two-step MR analysis was employed to dissect the correlation between TL and AN into two distinct phases. By utilizing brain white matter phenotypes as intermediaries, we separately investigated the causal relationships between TL and alterations in brain white matter phenotypes, and between changes in brain white matter phenotypes and AN [27,39,40]. 

To investigate the relationship between TL and alterations in brain white matter phenotypes, we employed a synergistic approach combining two-stage least squares (2SLS) and inverse-variance weighted (IVW) methods. This dual-method strategy aims to mitigate issues related to pleiotropy and IV validity, enhancing the robustness of our causal inference and enabling the identification of significantly associated phenotypes [27]. 

For exploring the association between changes in brain white matter phenotypes and AN, the IVW method was utilized under the assumption that all included IVs function as valid IVs. To further ensure the robustness of our findings, we conducted a sensitivity analysis using the weighted median approach, which provides robust estimates of causality, assuming that over 50% of the IVs are valid. This approach is applicable when estimating the effects of more than one IV. In cases where only a single IV was available, we employed the Wald ratio for principal component analysis [41].

### 2.4. Sensitivity Analysis

Sensitivity analysis included the scrutiny of heterogeneity and pleiotropy, alongside evaluating the influence of individual IVs on the aggregated outcomes of the MR analysis. Heterogeneity was assessed using MR–Egger methods, with the *Cochrane’s Q-test* employed for quantitative evaluation. Upon the detection of heterogeneity, a random effects model was deployed to recalibrate effect size estimates. Heterogeneity was further illustrated using scatter plots and funnel plots. Additionally, horizontal pleiotropy within IVs was investigated using the MR–Egger intercept method. This method helps identify any pleiotropic effects that could bias the results [42]. To assess the impact of individual IVs on the MR results, we employed the leave-one-out approach, which systematically excludes each IV one at a time to evaluate its influence on the overall findings [43].

### 2.5. Evaluation of Mediated Effects

To assess the overall impact of TL on AN, MR analyses were conducted on two independent samples. The overall effect of any exposure on the outcome can be decomposed into direct and indirect effects [27]. The direct effect of TL on AN was assessed by controlling for changes in the white matter phenotype. The indirect effect, or mediated effect, of TL on AN was computed as b1 × b2, where b1 represents the MR effect of TL on changes in the white matter phenotype, and b2 represents the MR effect of changes in the white matter phenotype on AN. Subsequently, standard errors for b1 and b2 were calculated using the Formula (2), and S values were obtained using the Sobel test. Z statistics and *p*-values were then calculated using Formulas (3) and (4). A *p*-value of less than 0.05 indicated a significant mediated effect [41].

(2) $$\;b_{m}=\sqrt{b_{1}\times s_{2}+b_{2}\times s_{1}}$$

(3) $$Z=\frac{b_{1}\times b_{2}}{S}$$

(4) $$95\%CI=b_{1}\times b_{2}+1.96S$$

## 3. Results

### 3.1. Direct MR Analysis and Sensitivity Analysis

**Figure 2:**
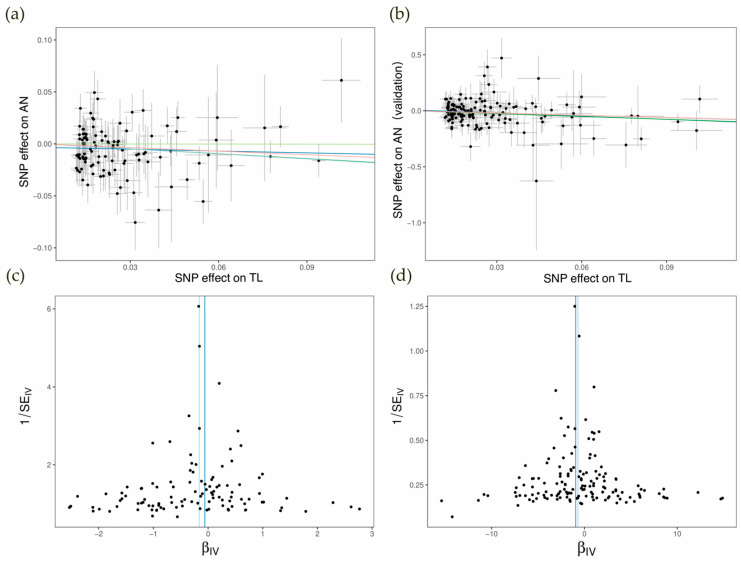
Scatter plots and funnel plots of direct Mendelian randomization (MR) analysis. (**a**) Scatter plot of causal relationship between telomere length (TL) and test group of anorexia nervosa (AN). (**b**) Scatter plot of causal relationship between TL and validation group of AN. (**c**) Funnel plot of causal relationship between TL and test group of AN. (**d**) Funnel plot of causal relationship between TL and validation group of AN.

To verify the direct causal relationship between TL and AN, we conducted a two-sample MR analysis. Following a series of quality control steps, including LDlink filtering, we identified 109 TL-related instrumental variables (IVs) in our test group. Instrument validity testing demonstrated that all variables in the MR analysis had sufficient strength, with F-statistics ranging from 29.85 to 1628.81 (Table S2). The IVW method showed a significant association between TL and AN (*p* < 0.05) (Table 1, Figure 2). To further confirm the relationship between TL and AN, after testing the instrument strength, we selected 144 IVs from external GWAS that met the filtering criteria (Table S2), which confirmed a strong negative correlation between TL and AN (Table 1). Our reverse Mendelian randomization study found no significant association between AN and TL (*p* > 0.05) (Table S3).

To validate the robustness of our direct effect results, we conducted sensitivity analyses to account for potential heterogeneity and pleiotropy. In the direct analysis of TL and AN, the results of pleiotropy tests and leave-one-out analysis indicated the robustness of the MR analysis results (*p* > 0.05); however, our results showed heterogeneity (*p* < 0.05). Nonetheless, the external validation passed all our sensitivity analyses (*p* > 0.05), including pleiotropy, heterogeneity tests, and leave-one-out analysis. External validation further confirmed the strong negative correlation between TL and AN, reinforcing our findings from the direct MR analysis. Detailed results of the sensitivity analyses are shown in Table S4, while the leave-one-out analysis results are depicted in Figure S1.

### 3.2. Two-Step MR Analysis

To investigate the mediating role of brain structure changes in the relationship between TL variation and increased risk of AN, we conducted a TSMR analysis, treating brain structure as the mediator.

**Step 1:** Relationship between TL and dMRI Phenotypes. We selected a subset of IVs from TL after quality control. Instrument validity tests indicated sufficient strength for all IVs in the MR analysis (F > 10) (Table S5). We performed MR analysis and 2SLS analysis between the selected IVs and 675 different dMRI phenotypes GWAS, finding significant correlations between TL and 208 dMRI phenotypes (2SLS: *p* < 0.05 and IVW: *p* < 0.05). After excluding the 44 GWAS datasets that did not pass the sensitivity analysis, we included the remaining 164 datasets in the next phase of the study. Detailed results of the first-step MR analysis are presented in Table S6, and the results of the 2SLS analysis are presented in Table S7. All scatter plots, funnel plots, and single SNP plots are shown in Figures S2–S4.

**Figure 3:**
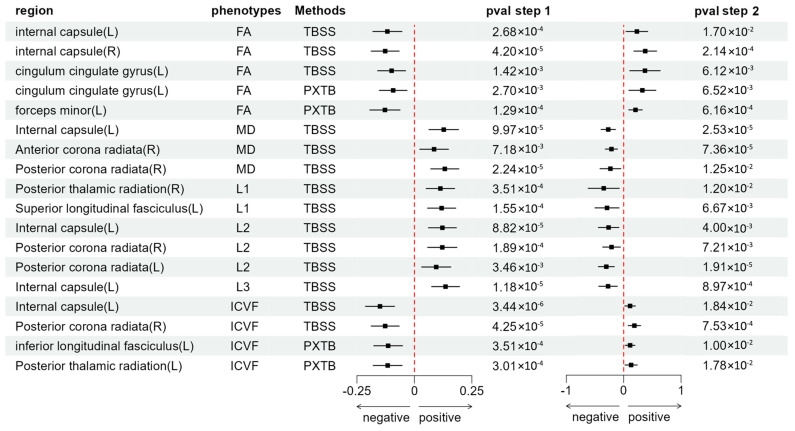
Forest plot of the results of two-step Mendelian randomization (MR) shows only the results where the final mediation effect is significant; Step 1: MR results between telomere length (TL) and dMRI phenotypes; Step 2: MR results between dMRI phenotypes and anorexia nervosa (AN).

**Step 2:** Relationship between dMRI Phenotypes and AN. To study the role of dMRI phenotypes in AN, we conducted another two-sample MR analysis to explore the potential causal relationship between dMRI phenotypes and AN. Due to our filtering criteria, some dMRI phenotypes GWAS could not filter IVs and were excluded (ubm-b-2044, ubm-b-2045 and ubm-b-2045). We selected the necessary IVs from the previous step’s GWAS according to the criteria, with instrument validity tests showing sufficient strength for all IVs (F > 10) (Table S8). MR analysis revealed significant associations between 49 dMRI phenotypes and AN (Wald ratio: *p* < 0.05 | IVW: *p* < 0.05). Detailed results of the second step MR analysis are presented in Table S9. All scatter plots, funnel plots, and single SNP plots are shown in Figures S5–S7. After conducting sensitivity analysis and mediation effect screening, the TSMR results for the 18 significant mediator phenotypes are presented in Figure 3. 

### 3.3. Two-Step Sensitivity Analysis

**Step 1:** Relationship between TL and dMRI Phenotypes. In the first part of the second stage, we verified the random relationship between TL and dMRI phenotypes. We found heterogeneity among GWAS with significant correlations; however, 44 dMRI phenotypes GWAS showed horizontal pleiotropy with TL (*p* < 0.05). To maintain the robustness of the final results, we excluded these GWAS before proceeding to the next MR step.

**Step 2:** Relationship between dMRI Phenotypes and AN. In the second part of the second stage, we excluded five significant heterogeneous results (*p* < 0.05) from the mediation effect discussion, all from our main dataset. We found no horizontal pleiotropy in the analysis. 

To verify the direction of our analysis, we used a Steiger analysis, selecting only the Steiger analysis results marked as true to ensure the correct analysis direction. All the sensitivity analysis details are provided in Table 2, Tables S10 and S11. We also used leave-one-out tests to examine our MR analysis. The MR results showed minimal change upon exclusion of specific SNPs, demonstrating the strong robustness of our methodology (Figures S8 and S9).

### 3.4. Calculation of Mediation Effect

**Figure 4:**
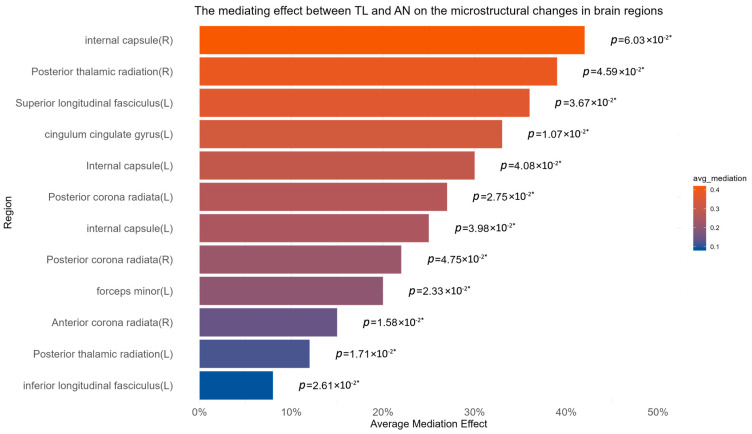
Bar chart of the average mediation effects for each brain region, incorporating only the 18 phenotypes selected for analysis. The average *p*-value for the mediation effects is indicated to the right of each bar, ensuring that each *p*-value is less than 0.05. Number with * indicates that the result is significant by our standards.

We examined the mediation effects of different mediators to validate the brain regions potentially mediating the TL–AN association. After the sensitivity analysis, we included 44 dMRI phenotypes GWAS results for a final mediation effect validation. Using the two-step MR results, we calculated the mediation effect values and significance, which are detailed in Table S12. After screening and excluding associations with non-significant mediation effects (*p* > 0.05), we obtained 18 strongly significant final results, which were all from the main dataset (Table 2, Figure 4). These findings highlight the specific brain regions where changes in the white matter microstructure mediate the relationship between TL and AN, providing critical insights into the neural mechanisms underlying the genetic influence of TL on the risk of developing AN.

## 4. Discussion

This study, through MR, sensitivity analysis, and external validation, demonstrated a significant one-way negative correlation between TL and AN. Previous research has shown a strong negative correlation between TL and age [43,44], suggesting that the shortening of TL with age may be associated with an increased incidence of AN [45]. It is worth noting that although our test group results showed heterogeneity, both our pleiotropy analysis and external validation supported a very stable negative correlation between TL and AN. Therefore, we still consider our results to be robust. To investigate the role of changes in brain white matter microstructure in this process, we selected 675 dMRI parameters of brain white matter as mediators and conducted TSMR and mediation effect analyses. Ultimately, we identified 18 strong and significant mediation effects. Our findings indicate that TL shortening is closely related to a series of white matter microstructural changes, which mediate an increased risk of AN, consistent with our initial hypothesis.

The results show that TL can alter AN risk by influencing the three eigenvalues (L1, L2, and L3) derived from the diffusion tensor in diffusion tensor imaging (DTI). DTI characterizes water molecule diffusion within tissues, and various dMRI phenotypes are calculated using these eigenvalues [46,47,48]. In parts of the internal capsule, cingulate gyrus, and forceps minor, TL is positively correlated with the FA phenotype, while FA is negatively correlated with AN. An increase in FA indicates enhanced water molecule diffusion along a primary direction, usually associated with well-structured and directionally consistent fiber tracts. This suggests that as age increases and TL shortens, the fiber tract structures in these brain regions are disrupted, potentially increasing the risk of AN.

In parts of the internal capsule and corona radiata, TL is positively correlated with the MD phenotype, while MD is positively correlated with AN. MD is the average of the diffusion coefficients along the three axes of the water diffusion model (L1, L2, L3) and is commonly used to assess tissue health. A high MD value may be associated with white matter damage, inflammation, or lesions [49,50]. Our results indicate that as TL shortens, the overall diffusion capacity of water in these brain regions may increase, implying that normal white matter microstructure is disrupted, potentially increasing the risk of AN. Additionally, we found that in parts of the internal capsule, corona radiata, inferior longitudinal fasciculus, and thalamic radiation, TL is positively correlated with the ICVF phenotype. ICVF, derived from neurite orientation dispersion and density imaging, represents the intracellular volume fraction within the tissue (e.g., neurons, glial cells). A low ICVF value may indicate nerve fiber damage or reduced neuron density [51,52,53]. The results suggest that as age increases and TL shortens, nerve fibers in these brain regions may degenerate, or neuron density may decrease, potentially increasing the risk of AN.

Numerous early studies have reported a direct association between TL and neurological disorders, including depression, anxiety, Alzheimer’s disease, and Parkinson’s disease [54,55]. Merete Osler et al. reported a negative correlation between TL and psychiatric disorders, such as post-traumatic stress disorder, anxiety disorders, and depression [56]. However, the causal relationship between TL and AN has not been clearly elucidated. Annya M. Smyth et al. discussed the association of biological abnormalities in schizophrenia (e.g., inflammation, oxidative stress, and changes in steroid or biogenic amine activity) with telomere shortening, but understanding and application of telomere shortening in this context remain incomplete due to these abnormalities crossing traditional psychiatric diagnoses [57,58]. TL shortening may be associated with specific biological processes or endophenotypes and may also be linked to specific diagnostic categories. This hypothesis requires thorough testing, as it could help explain inconsistencies in TL results within specific diagnostic groups and the heterogeneity observed between different diagnostic groups. Uziel et al. conducted an observational study that collected samples from adolescent females and found “no difference in TL between AN patients and controls” [59]. This may be due to insufficient sample sizes across different age groups, including TL data from 44 female adolescent AN patients at admission, 18 patients at discharge, and 22 control patients. Additionally, our study found that the effect of TL on AN is unidirectional. Moreover, a shorter disease course and insufficient reduction in BMI may also influence the appearance of TL shortening, leading to a discrepancy between their findings and ours. Suda et al.’s study explored the association between changes in connectivity strength in certain brain regions and AN, but this association does not imply causality [60]. Our research, on the other hand, focused on 675 effective dMRI phenotypes and used genetic-variation-based instrumental variables to investigate the causal impact of aging-related genetic variations on brain function and corresponding white matter. Furthermore, we evaluated the causal effect of these genetic variations on the onset of AN.

The findings presented in this study suggest that TL may influence AN by mediating imbalances in white matter microstructure. AN also does not have a reverse effect on TL. Certain psychiatric disorders may also be associated with other factors influencing TL (e.g., sleep deprivation, malnutrition, physical inactivity, smoking), which are not directly causally related to the disease. This aligns with the viewpoint that lifestyle changes typically accompanying certain psychiatric disorders may secondarily lead to TL shortening [61]. These studies reveal the relationship between TL and neurodegenerative changes in brain structure. Building on previous research, our study further demonstrated a significant negative correlation between TL and AN, reporting an increased risk of AN as TL shortens.

A cross-sectional study involving MRI scans reported that TL is associated only with the volumes of certain subregional areas, such as the hippocampus, amygdala, precuneus, thalamus, and ventral diencephalon [62]. Additionally, a Swedish study involving 57 middle-aged women demonstrated that shortened TL is associated with reduced hippocampal volume, with a strong relationship in APOE ε4 non-carriers but less clear in ε4 carriers [63]. A recent meta-analysis suggested that a longer leukocyte TL is associated with total brain and hippocampal volume, but not with white matter hyperintensities (WMHs) [64]. In contrast, a two-year follow-up study of elderly individuals found that extremely short TL is associated with faster cognitive decline and the progression from mild cognitive impairment to Alzheimer’s disease [65]. Existing research has identified common genomic causal loci affecting TL, brain morphology, and genetic regulatory traits [66]. The identified genes are involved in the central nervous system, providing evidence that shared genetics can partly explain the associations between TL and several previously reported brain-based outcomes. These results emphasize that TL can serve as a biomarker for certain brain-based diseases, such as psychiatric disorders. Furthermore, these findings confirm that telomere-based cellular aging, in addition to methylation-based biological aging, has the potential to further elucidate the biological mechanisms of brain diseases [67].

The most consistent findings show a positive correlation between TL and total brain volume and hippocampal volume [68,69]. These associations are relevant to disease pathogenesis, as overall atrophy and hippocampal atrophy are characteristics of Alzheimer’s disease. A small trial on psychological training aimed at cultivating presence, compassion, and social cognitive skills found that longitudinal changes in TL are associated with changes in cortical thickness [70]. However, it remains unclear how TL relates to other structural and functional brain measures related to neurological health [71]. These include gray matter structure, white matter microstructure, and functional connectivity. MRI endophenotypes are quantitative and, in the disease pathogenic pathway, are closer to the fundamental genetic determinants compared to clinical phenotypes [72]. Establishing the relationship between TL and MRI biomarkers can provide insights into the biological mechanisms of neurodegenerative diseases. Building on previous research, our study further demonstrates the causal relationship between TL changes and white matter microstructural changes through a Mendelian randomization analysis of TL data and dMRI phenotypes. This lays a solid foundation for exploring the potential mechanisms underlying age-related psychiatric disorders.

Previous DTI analyses have shown that, compared to healthy controls, women with AN exhibit increased FA in the right corticospinal projections and lingual gyrus, while FA in the corpus callosum, left superior longitudinal fasciculus (SLF), and precentral gyrus is significantly decreased [73]. One neuroimaging study found that patients with AN have reduced volume in the middle temporal gyrus (MTG). A functional MRI study also indicated significantly reduced activation in the MTG of an AN patient during self–other body size comparisons. Studies on current AN patients reveal extensive gray matter reductions in neocortical areas and regions associated with emotional regulation and reward (e.g., anterior cingulate cortex, orbitofrontal cortex, insular cortex, hippocampus and parahippocampus, amygdala, and striatum) [74,75,76]. Other studies, however, report increased gray matter in the neocortex and limbic regions [77,78]. Despite some of these changes potentially normalizing in recovered patients, other research indicates that volume changes may persist in recovered individuals [79,80]. These changes’ distribution suggests disruptions in inter-regional brain connectivity, similar to what is observed in most other psychiatric disorders [81,82]. Therefore, degeneration of the white matter microstructure in the brain, which connects various regions, may directly or indirectly lead to an increased risk of AN.

Alterations in the structure of the SLF correspond with gray matter reductions in the frontal and temporoparietal regions of AN patients and are consistent with some results from previous DTI studies on AN [83]. The findings in the left SLF seem functionally related to one of the characteristics of AN: visual self-recognition distortion. The medial and inferior parietal regions are involved in processes such as proprioception, size and spatial judgment, visual imagery, and integration of visual information, which are all neural processes underpinning body self-image representation [84]. In turn, body image perception is integrated into functional networks connecting prefrontal and parietal regions, with the SLF being the primary white matter tract connecting these areas. Consequently, some studies have found differences in the activation of the parietal cortex and prefrontal regions between AN patients and controls when visualizing their own body image [85]. Building on previous research, our study further explores the relationship between brain white matter structure and AN, identifying a causal relationship between degenerative changes in the internal capsule, corona radiata, posterior thalamic radiation, left cingulate gyrus, SLF, and left tapetum with AN. Our findings are different but do not conflict with the idea proposed by Griffiths et al.. that “the changes in white matter microstructure observed in patients with AN can indeed be reversed by weight recovery”. We found that aging may lead to potential changes in white matter microstructure, thus affecting the onset of AN. Aging is a macro and irreversible change, and the reverse effect of weight recovery on the destruction of white matter microstructure is relatively limited, which suggested that there is a dynamic relationship between white matter integrity and the pathology of AN that may impact our understanding of the long-term and aging effects of these changes. As we explored in studies linking TL to AN risk and involving specific brain regions [86], we demonstrated that degenerative changes in the SLF could indeed increase the risk of AN by altering connectivity between gray matter regions. Additionally, we hypothesize that AN risk resulting from changes in other brain regions with aging is systemic, with the underlying mechanisms warranting further investigation.

The advantage of MR lies in its ability to overcome confounding factors and reverse causation issues that are unavoidable in traditional observational studies [34]. Selecting appropriate IVs is a crucial step to ensure the validity and reliability of MR analysis. In this study, based on the requirements of MR analysis, we selected genetic variants strongly associated with TL and 675 dMRI phenotypes from a large genomic database, ensuring the independence and pleiotropy-free nature of these IVs. Additionally, we calculated F-statistics to evaluate the strength of the IVs, ensuring that the genetic variants provided sufficient statistical power for reliable causal inference.

In practice, we selected disease populations unrelated to the exposure group as the outcome group to assess the causal effects across different population structures from various databases. To validate the robustness of the MR analysis, we performed sensitivity analyses and employed different MR methods to comprehensively assess the validity of causal estimates, thereby enhancing the robustness of the results [87]. We used the intercept test from MR–Egger regression to evaluate potential pleiotropy bias and Cochran’s Q statistic to assess the heterogeneity of the results, ensuring consistency among different IVs. With no significant results in the sensitivity analysis, we could assess the robustness of the main analysis results, ensuring that the study conclusions were not affected by potential statistical bias or data issues [88,89]. Overall, the MR methods employed in this study, the screening and quality control of IVs, and the implementation of sensitivity analyses all adhered to the standard guidelines for MR analysis [28]. Our findings not only deepen our understanding of the causal relationship between TL and AN but also provide a solid theoretical basis for further understanding the mediating effects of white matter microstructural changes and for developing prevention and treatment strategies.

Despite the robust analytical tool that MR provides for exploring the potential causal relationship between TL and AN, this research field still faces numerous challenges. Firstly, AN is a complex disease influenced by multiple genes and environmental factors. The involvement of numerous genetic variants, each with relatively small effects on the disease, increases the complexity of the study and requires large sample sizes to enhance statistical power [90]. Secondly, while some genetic variants have shown associations with AN, whether these variants directly influence AN risk requires further verification [91]. Furthermore, although the fundamental assumption of MR analysis is that genetic variants remain fixed throughout an individual’s life and are unaffected by environment and lifestyle, the biological effects of genetic variants can be influenced by environmental factors, individual lifestyle, and interactions with other genetic factors [92]. These interactions may interfere with the interpretation of MR analysis results as they can alter the impact of genetic variants on disease risk.

To further understand the causal relationships between TL, white matter microstructure, and AN, and to promote the development of effective prevention and treatment strategies, future research needs to expand in several areas. More methods should be employed to verify and detail the specific mechanisms of TL and the white matter microstructure in the formation of AN, such as multimodal imaging techniques [93], genetic analyses [94,95,96], epigenetic studies [97,98,99], and bioinformatics technologies [100,101]. By integrating these advanced methodologies, we can obtain a more comprehensive understanding of the complex interplay between genetic and environmental factors in AN. This will ultimately lead to the development of more targeted and effective interventions aimed at mitigating the risk of AN by preserving TL and maintaining the integrity of the brain’s white matter microstructure.

## 5. Conclusions

Our study demonstrated a significant negative correlation between TL and AN through an MR analysis. Additionally, it revealed that changes in specific white matter microstructures, such as the internal capsule, corona radiata, posterior thalamic radiation, left cingulate gyrus, left longitudinal fasciculus, and left forceps minor, may mediate this correlation. These findings enhance our understanding of the neural mechanisms underlying the exacerbation of AN with aging, highlight the role of brain functional networks in disease progression, and provide potential biological targets for future therapeutic interventions aimed at treating AN.

## Figures and Tables

**Table 1:** Mendelian randomization (MR) results of telomere length (TL) on anorexia nervosa (AN) (Test and Validation).

Method	Test				Validation			
	Nsnp	*p*-Value	OR	95%CI	Nsnp	*p*-Value	OR	95%CI
MR–Egger	109	6.35 × 10^−1^	9.43 × 10^−1^	0.74–1.20	144	4.90 × 10^−2^	3.92 × 10^−1^	0.16–0.99
Weighted median	109	1.21 × 10^−1^	8.54 × 10^−1^	0.70–1.04	144	4.94 × 10^−2^	4.31 × 10^−1^	0.19–1.00
IVW	109	1.74 × 10^−2 a^	8.51 × 10^−1^	0.75–0.97	144	7.36 × 10^−3 b^	5.02 × 10^−1^	0.30–0.83
Simple mode	109	9.89 × 10^−1^	9.97 × 10^−1^	0.67–1.48	144	4.48 × 10^−1^	5.18 × 10^−1^	0.10–2.82
Weighted mode	109	2.91 × 10^−1^	8.91 × 10^−1^	0.72–1.10	144	2.13 × 10^−1^	5.18 × 10^−1^	0.19–1.45

^a^ The *p*-value of the MR analysis performed between TL and the test group of AN using the IVW method shows significant casual relationship. The p-value for the heterogeneity test was 0.023, and the *p*-value for the horizontal pleiotropy test was 0.330. ^b^ The *p*-value of the MR analysis performed between TL and the validation group of AN using the IVW method shows significant causal relationship. The p-value for the heterogeneity test was 0.587, and the *p*-value for the horizontal pleiotropy test was 0.532. Nsnp, number of single nucleotide polymorphisms; OR, odds ratio; 95% CI, 95% confidence interval; IVW, inverse-variance weighted.

**Table 2:** Two-step sensitivity analysis results of 18 significant mediator phenotypes. p_pe_, *p*-value of two-stage least squares; MRSr, Multiple R-squared of two-stage least squares; p_het,_ *p*-value of the heterogeneity test; p_ple_, *p*-value of the horizontal pleiotropy test; p_steiger_, *p*-value of the Steiger analysis.

dMRI Phenotypes			Step1					Step2		
Region	Phenotypes	Methods	P_pe_	MRSr	p_het_	p_ple_	p_steiger_	p_het_	p_ple_	p_steiger_
Internal capsule (L)	FA	TBSS	4.68 × 10^−8^	2.04 × 10^−1^	3.40 × 10^−1^	6.10 × 10^−2^	1.37 × 10^−65^	2.23 × 10^−1^	9.19 × 10^−1^	1.19 × 10^−55^
Internal capsule (R)	FA	TBSS	8.12 × 10^−7^	1.69 × 10^−1^	4.44 × 10^−1^	6.25 × 10^−2^	2.50 × 10^−71^	5.33 × 10^−1^	9.64 × 10^−1^	3.80 × 10^−31^
Cingulum cingulate gyrus (L)	FA	TBSS	1.67 × 10^−3^	7.46 × 10^−2^	9.75 × 10^−1^	2.71 × 10^−1^	7.05 × 10^−77^	1.23 × 10^−1^	9.45 × 10^−1^	6.87 × 10^−41^
Cingulum cingulate gyrus (L)	FA	PXTB	6.54 × 10^−4^	8.39 × 10^−2^	8.57 × 10^−1^	8.69 × 10^−1^	3.86 × 10^−80^	6.87 × 10^−1^	8.89 × 10^−1^	5.56 × 10^−28^
Forceps minor (L)	FA	PXTB	8.81 × 10^−4^	8.19 × 10^−2^	1.59 × 10^−1^	1.13 × 10^−1^	1.01 × 10^−64^	5.85 × 10^−1^	8.31 × 10^−1^	6.40 × 10^−127^
Internal capsule (L)	MD	TBSS	1.84 × 10^−3^	7.22 × 10^−2^	2.27 × 10^−1^	9.32 × 10^−2^	4.10 × 10^−57^	4.43 × 10^−1^	9.69 × 10^−1^	1.78 × 10^−97^
Anterior corona radiata (R)	MD	TBSS	2.80 × 10^−2^	3.77 × 10^−2^	8.16 × 10^−1^	3.40 × 10^−1^	5.94 × 10^−65^	7.11 × 10^−1^	8.50 × 10^−1^	4.17 × 10^−147^
Posterior corona radiata (R)	MD	TBSS	3.94 × 10^−4^	9.52 × 10^−2^	8.97 × 10^−1^	8.95 × 10^−1^	6.33 × 10^−67^	8.03 × 10^−2^	5.57 × 10^−1^	2.48 × 10^−85^
Posterior thalamic radiation (R)	L1	TBSS	6.02 × 10^−3^	5.84 × 10^−2^	4.05 × 10^−1^	1.15 × 10^−1^	1.71 × 10^−63^	1.19 × 10^−1^	9.15 × 10^−1^	1.76 × 10^−33^
Superior longitudinal fasciculus (L)	L1	TBSS	5.45 × 10^−5^	1.24 × 10^−1^	7.23 × 10^−1^	9.67 × 10^−1^	8.76 × 10^−68^	5.62 × 10^−2^	6.35 × 10^−1^	4.48 × 10^−68^
Internal capsule (L)	L2	TBSS	7.95 × 10^−7^	1.72 × 10^−1^	5.22 × 10^−1^	5.52 × 10^−2^	4.45 × 10^−63^	2.97 × 10^−1^	7.89 × 10^−1^	2.34 × 10^−49^
Posterior corona radiata (R)	L2	TBSS	1.27 × 10^−5^	1.39 × 10^−1^	3.17 × 10^−1^	7.28 × 10^−1^	4.34 × 10^−63^	8.45 × 10^−1^	9.72 × 10^−1^	5.04 × 10^−79^
Posterior corona radiata (L)	L2	TBSS	4.00 × 10^−4^	9.43 × 10^−2^	3.73 × 10^−1^	3.41 × 10^−1^	1.91 × 10^−61^	6.40 × 10^−1^	7.81 × 10^−1^	2.18 × 10^−84^
Internal capsule (L)	L3	TBSS	4.16 × 10^−4^	9.17 × 10^−2^	6.08 × 10^−1^	8.96 × 10^−2^	6.53 × 10^−70^	1.46 × 10^−1^	6.29 × 10^−1^	2.26 × 10^−84^
Internal capsule (L)	ICVF	TBSS	2.14 × 10^−5^	1.33 × 10^−1^	2.80 × 10^−1^	9.49 × 10^−2^	1.25 × 10^−58^	2.25 × 10^−1^	8.52 × 10^−2^	1.66 × 10^−233^
Posterior corona radiata (R)	ICVF	TBSS	1.72 × 10^−2^	4.26 × 10^−2^	6.93 × 10^−1^	7.17 × 10^−1^	2.80 × 10^−70^	4.45 × 10^−1^	7.87 × 10^−1^	3.60 × 10^−147^
inferior longitudinal fasciculus (L)	ICVF	PXTB	2.13 × 10^−4^	1.03 × 10^−1^	6.40 × 10^−1^	5.90 × 10^−2^	4.33 × 10^−63^	1.95 × 10^−1^	6.90 × 10^−1^	7.95 × 10^−259^
Posterior thalamic radiation (L)	ICVF	PXTB	1.05 × 10^−3^	8.40 × 10^−2^	7.33 × 10^−1^	5.29 × 10^−2^	1.28 × 10^−64^	9.23 × 10^−2^	2.79 × 10^−1^	2.02 × 10^−183^

## Data Availability

The data presented in this study are openly available in openGWAS, reference number [20,24,25], and FinnGen database at [10.1038/s41586-022-05473-8], reference number [26].

## Supplementary Materials

The following supporting information can be downloaded at: https://www.mdpi.com/article/10.3390/biomedicines12081874/s1, Table S1: Details of the selected 675 dMRI phenotypic data. Table S2: Instrumental variables (IVs) selected for direct MR analysis. Table S3: selected IVs and results for reverse MR analysis. Table S4: Detailed results of the sensitivity analyses for direct MR analysis. Table S5: IVs selected for two-step Mendelian Randomization (TSMR) analysis (STEP 1). Table S6: Detailed results of TSMR analysis (STEP 1). Table S7: Detailed results of the 2SLS analyses for TSMR analysis (STEP 1). Table S8: IVs selected for TSMR analysis (STEP 2). Table S9: Detailed results of TSMR analysis (STEP 2). Table S10: Detailed results of the sensitivity analyses for TSMR analysis (STEP 1). Table S11: Detailed results of the sensitivity analyses for TSMR analysis (STEP 2). Table S12: Mediation effect calculation results of significant results that passed sensitivity analysis. Figure S1: Leave-one-out analysis results of direct MR analysis. Figure S2: Scatter plots of TSMR analysis (STEP 1). Figure S3: Funnel plots of TSMR analysis (STEP 1). Figure S4: Single SNP plots of TSMR analysis (STEP 1). Figure S5: Scatter plots of TSMR analysis (STEP 2). Figure S6: Funnel plots of TSMR analysis (STEP 2). Figure S7: Single SNP plots of TSMR analysis (STEP 2). Figure S8: Leave-one-out analysis results of TSMR analysis (STEP 1). Figure S9: Leave-one-out analysis results of TSMR analysis (STEP 2).

## Appendix A

**Table A1:** Detailed descriptions of the phenotypes of dMRI parameters.

Name of Parameter	Brief Description	Common Interpretation
Fractional anisotropy (FA)	A scalar value describing the degree of anisotropy of a diffusion process, ranging from 0 (isotropic diffusion, no directionality) to 1 (highly anisotropic diffusion, strong directionality).	Commonly used to assess white matter integrity. Higher FA values are associated with greater fiber density, axonal diameter, and myelination. Lower FA values can indicate white matter damage or degeneration [102].
Intra-Cellular Volume Fraction (ICVF)	The fraction of water diffusion restricted within intra-neurite compartments, reflecting neuronal density and dendritic complexity.	Changes in ICVF can reflect pathological conditions such as neuroinflammation or neurodegeneration. Higher ICVF values suggest greater cellular density, which can be indicative of healthy neural tissue [103].
Isotropic Volume Fraction (ISOVF)	The fraction of water diffusion that is isotropic or hindered within tissues.	Higher ISOVF values may indicate increased tissue damage, edema, or isotropic diffusion due to reduced tissue integrity [104].
Principal eigenvalue or axial diffusivity (L1)	The magnitude of water diffusion along the primary direction within a voxel.	Changes in axial diffusivity can indicate axonal injury or alterations in myelin sheath integrity, providing specific information on white matter pathology [105].
Secondary eigenvalue (L2)	The magnitude of water diffusion perpendicular to the principal direction.	Secondary eigenvalue changes can reflect alterations in tissue integrity and may be indicative of more diffuse tissue damage, particularly in regions with complex fiber orientations [105].
Tertiary eigenvalue (L3)	The magnitude of water diffusion in the direction with the least diffusion.	Like the secondary eigenvalue, changes in the tertiary eigenvalue can help characterize the microstructural complexity of the tissue, especially in areas with crossing fibers [105].
Mean diffusivity (MD)	The average magnitude of water diffusion within a voxel, irrespective of direction.	Increased MD values are generally associated with tissue pathology such as cell swelling, edema, or decreased cell density, while decreased MD may suggest increased tissue organization or coherence [106].
Mode of Anisotropy (MO)	The shape of diffusion tensor ellipsoids within tissues.	Different modes of anisotropy (e.g., planar, linear, spherical) provide insights into tissue microstructure changes and organization, aiding in the characterization of white matter tracts and pathology [102].
Orientation Dispersion (OD)	The variability in fiber orientation within a voxel.	Higher orientation dispersion values indicate less coherent fiber orientations within a voxel, which may occur in regions with crossing fibers or disrupted white matter architecture [107].
