# Supplementary figures and images for: Causal Relationship between Aging and Anorexia Nervosa: A White-Matter-Microstructure-Mediated Mendelian Randomization Analysis
Source: Biomedicines. 2024 Aug 16;12(8):1874. doi: 10.3390/biomedicines12081874 (PMC11351342; doi:10.3390/biomedicines12081874)

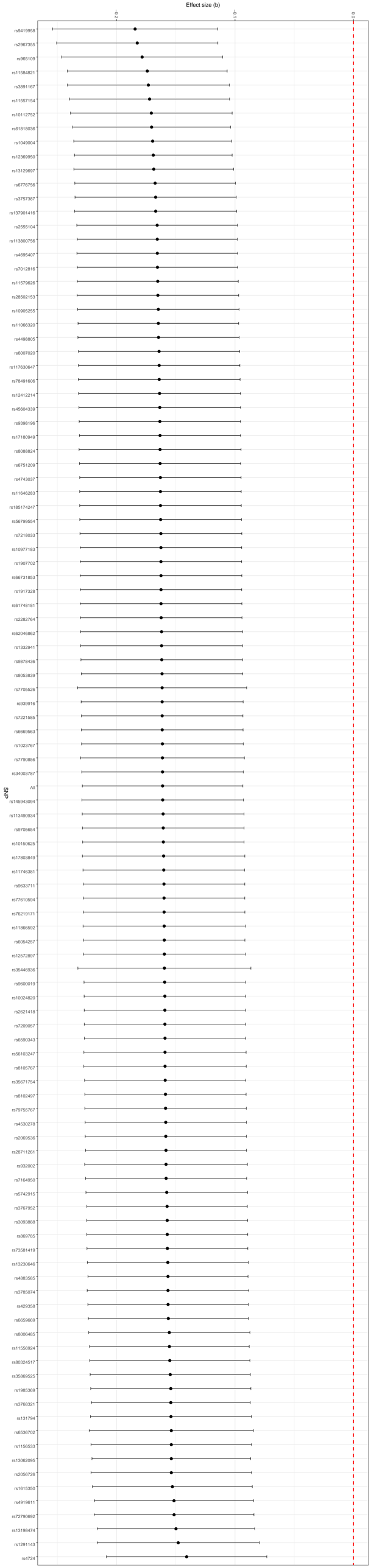

Supplement: Supplementary file 1 [file biomedicines-12-01874-s001.zip › Supplementary Figure/Figure S1/Leaveoneout plot of MR analysis for TL to test group.pdf]

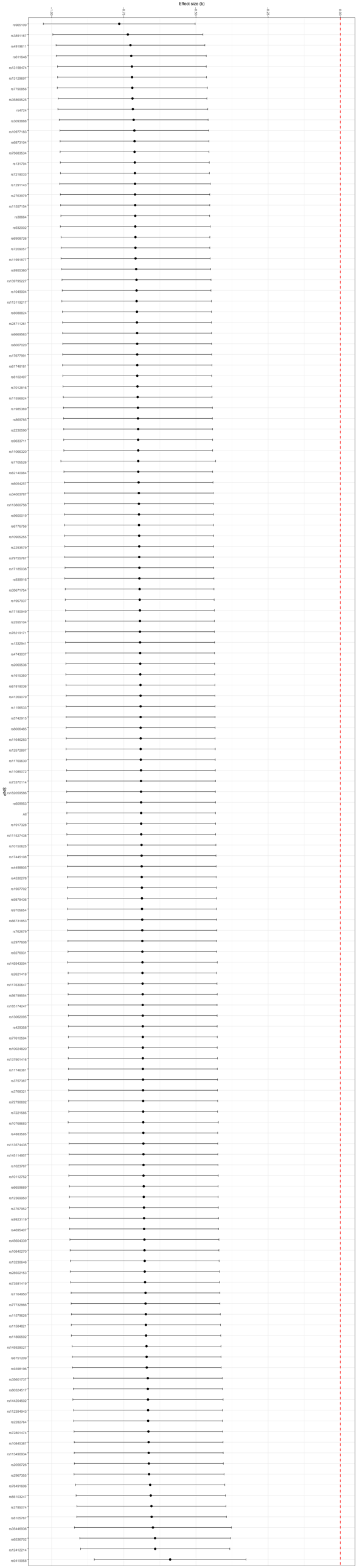

Supplement: Supplementary file 1 [file biomedicines-12-01874-s001.zip › Supplementary Figure/Figure S1/Leaveoneout plot of MR analysis for TL to validate group.pdf]

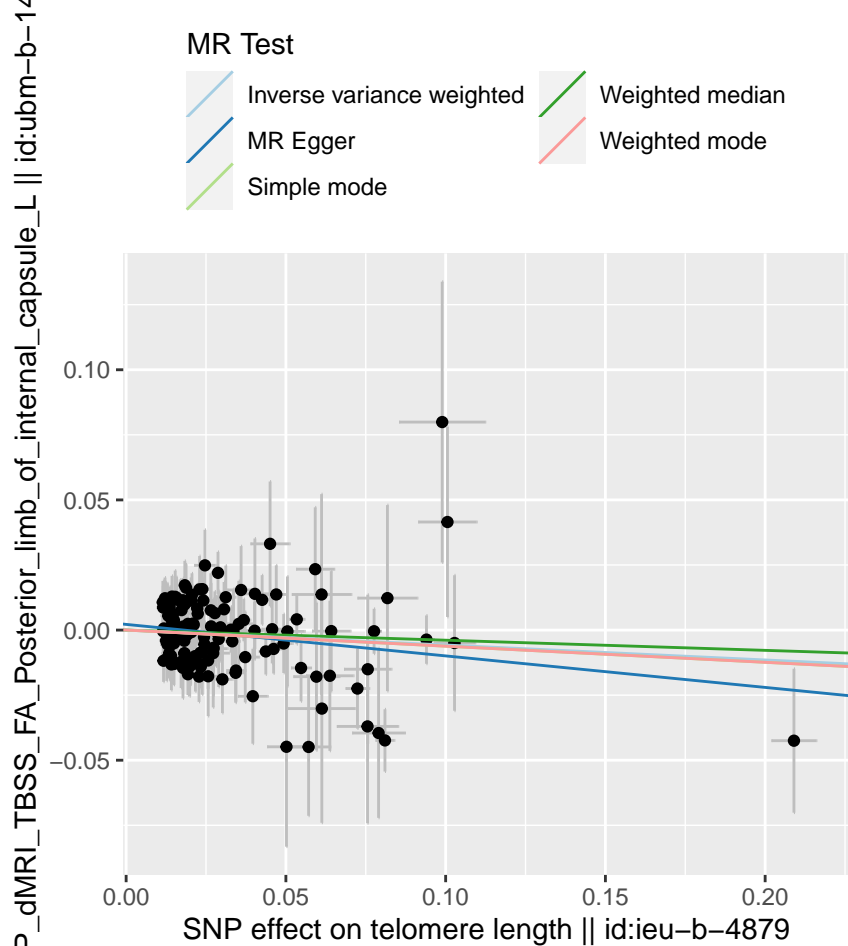

Supplement: Supplementary file 1 [file biomedicines-12-01874-s001.zip › Supplementary Figure/Figure S2/Scatter plot of MR analysis for TL to ubm-b-1471.pdf]

# MR Test

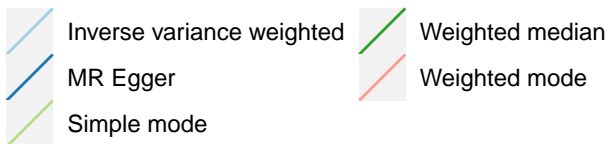

effect on IDP\_dMRI\_TBSS\_FA\_Sagittal\_stratum\_R || id:ubm-b-1482

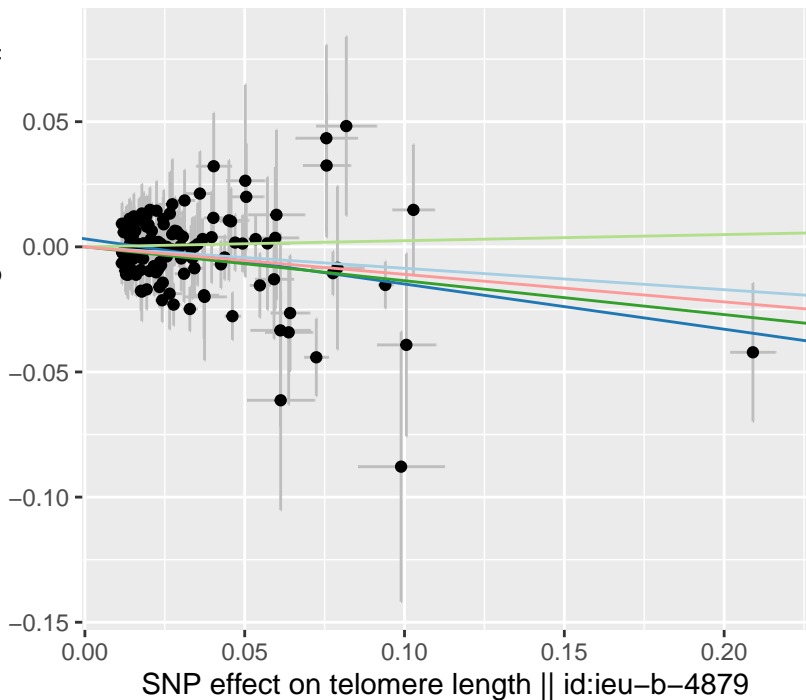

Supplement: Supplementary file 1 [file biomedicines-12-01874-s001.zip › Supplementary Figure/Figure S2/Scatter plot of MR analysis for TL to ubm-b-1482.pdf]

# MR Test

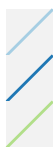

Inverse variance weighted

MR Egger

Simple mode

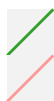

Weighted median

Weighted mode

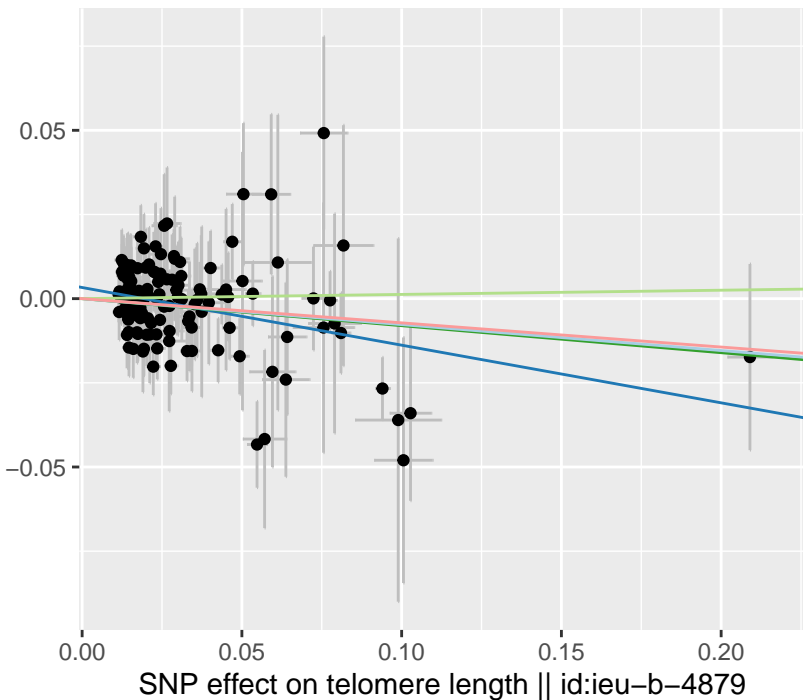

Supplement: Supplementary file 1 [file biomedicines-12-01874-s001.zip › Supplementary Figure/Figure S2/Scatter plot of MR analysis for TL to ubm-b-1486.pdf]

# MR Test

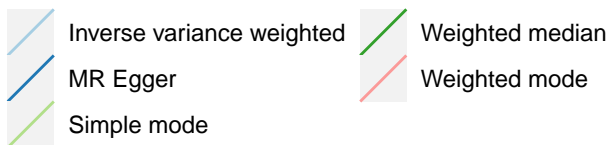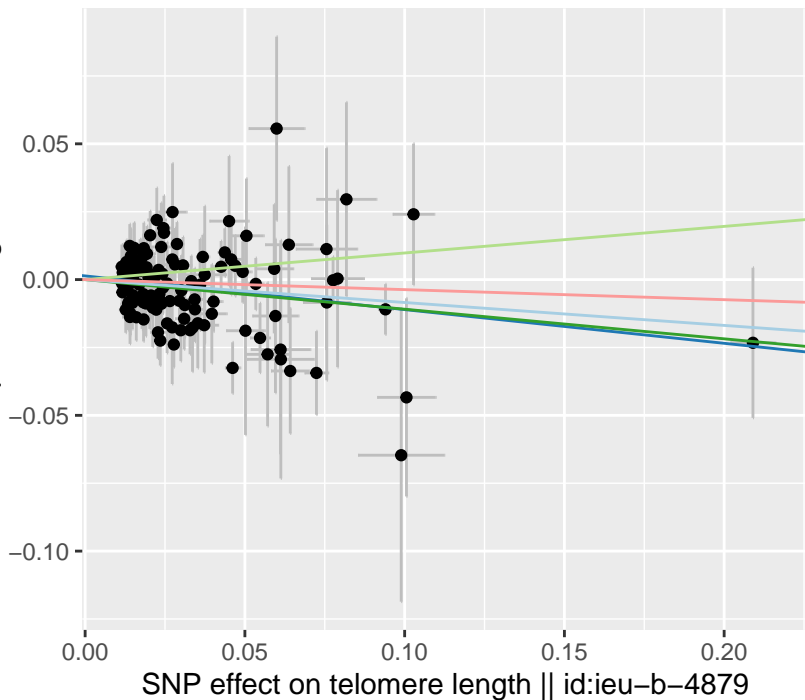

Supplement: Supplementary file 1 [file biomedicines-12-01874-s001.zip › Supplementary Figure/Figure S2/Scatter plot of MR analysis for TL to ubm-b-1492.pdf]

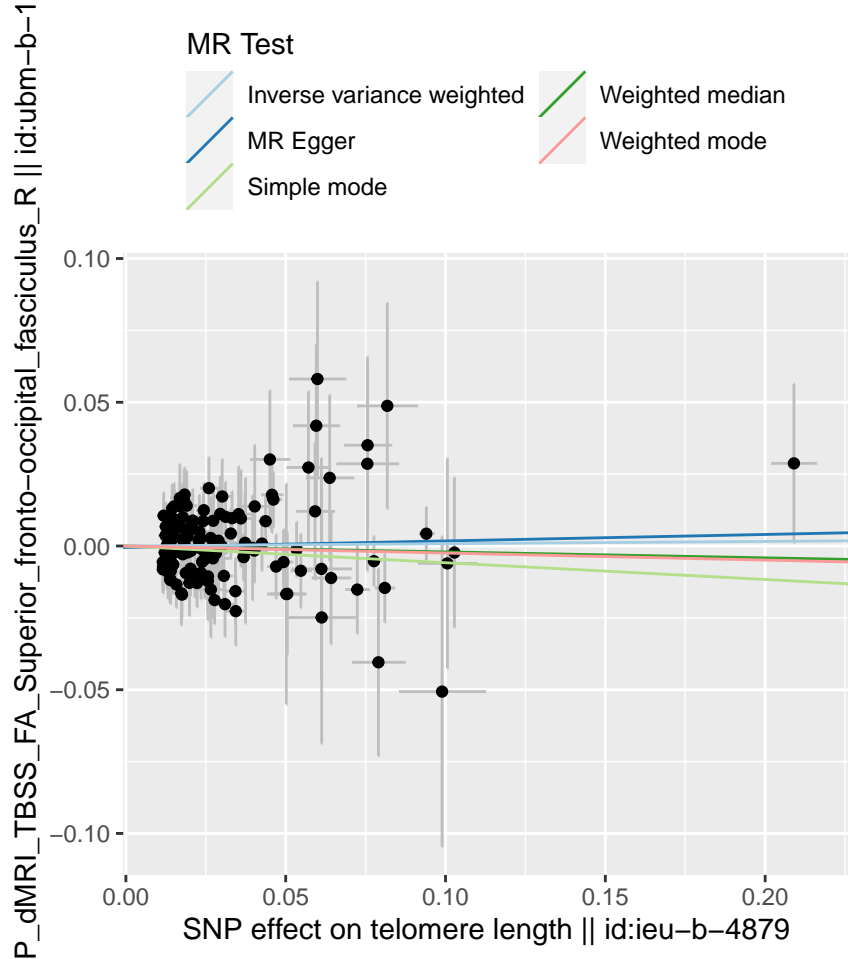

Supplement: Supplementary file 1 [file biomedicines-12-01874-s001.zip › Supplementary Figure/Figure S2/Scatter plot of MR analysis for TL to ubm-b-1494.pdf]

# MR Test

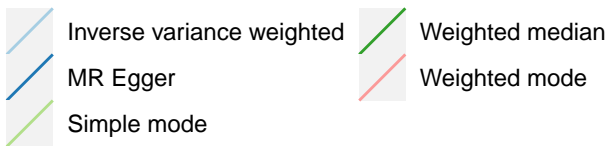

NP effect on IDP\_dMRI\_ProbtrackX\_FA\_slf\_I || id:ubm-b-1521

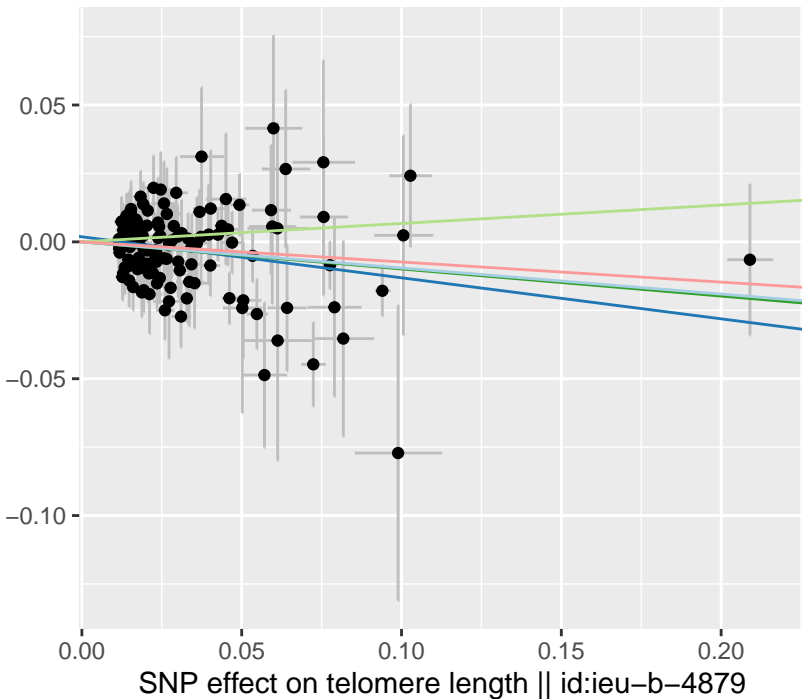

Supplement: Supplementary file 1 [file biomedicines-12-01874-s001.zip › Supplementary Figure/Figure S2/Scatter plot of MR analysis for TL to ubm-b-1521.pdf]

## MR Test

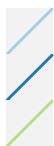

Inverse variance weighted

MR Egger

Simple mode

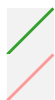

Weighted median

Weighted mode

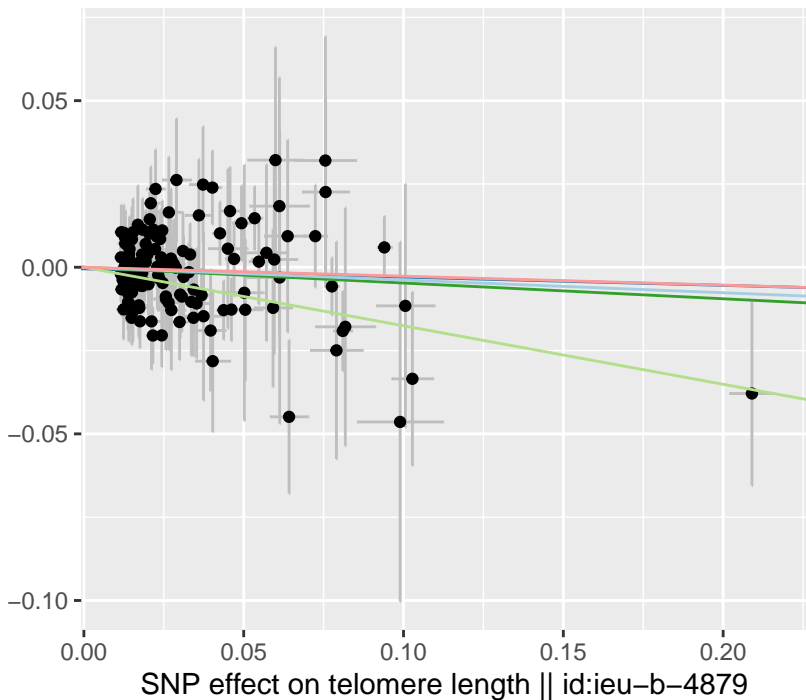

Supplement: Supplementary file 1 [file biomedicines-12-01874-s001.zip › Supplementary Figure/Figure S2/Scatter plot of MR analysis for TL to ubm-b-1540.pdf]

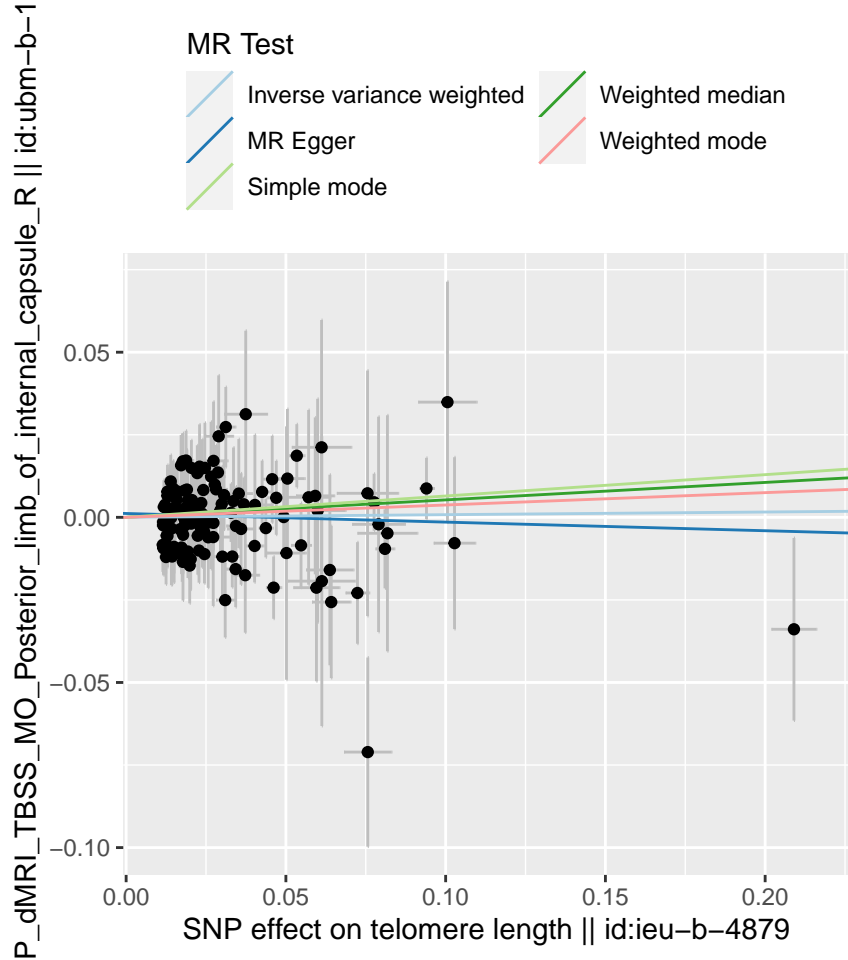

Supplement: Supplementary file 1 [file biomedicines-12-01874-s001.zip › Supplementary Figure/Figure S2/Scatter plot of MR analysis for TL to ubm-b-1545.pdf]

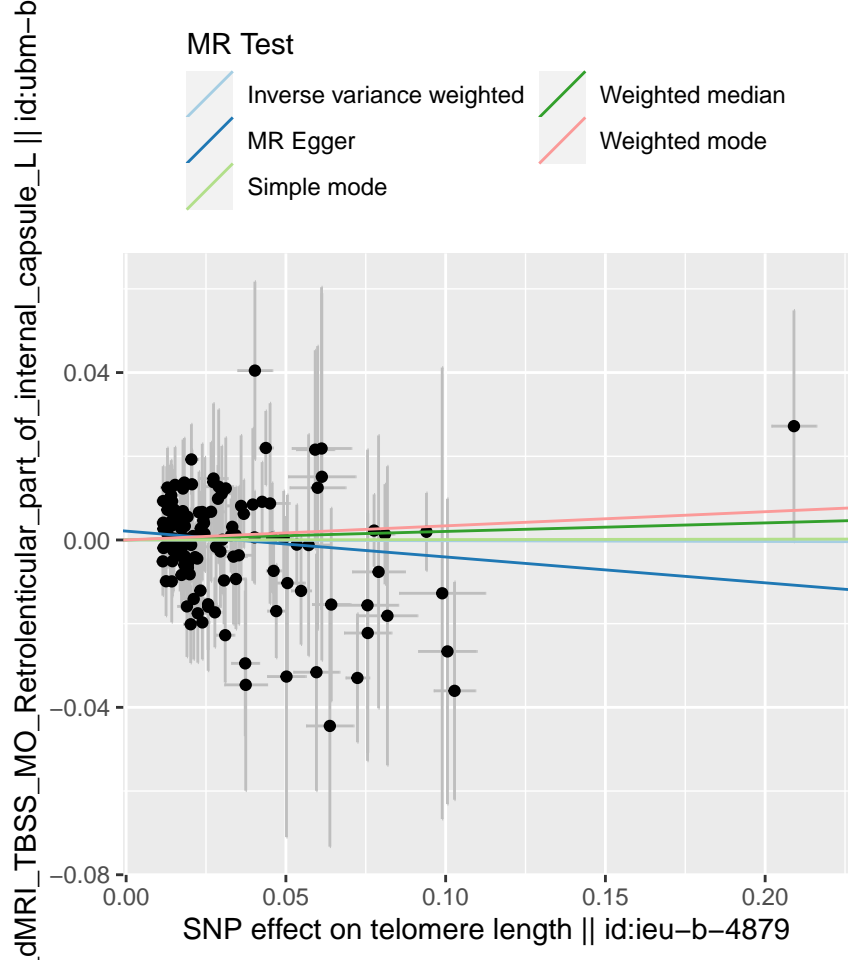

Supplement: Supplementary file 1 [file biomedicines-12-01874-s001.zip › Supplementary Figure/Figure S2/Scatter plot of MR analysis for TL to ubm-b-1548.pdf]

# MR Test

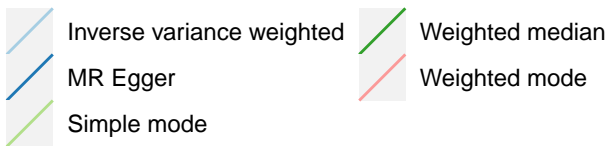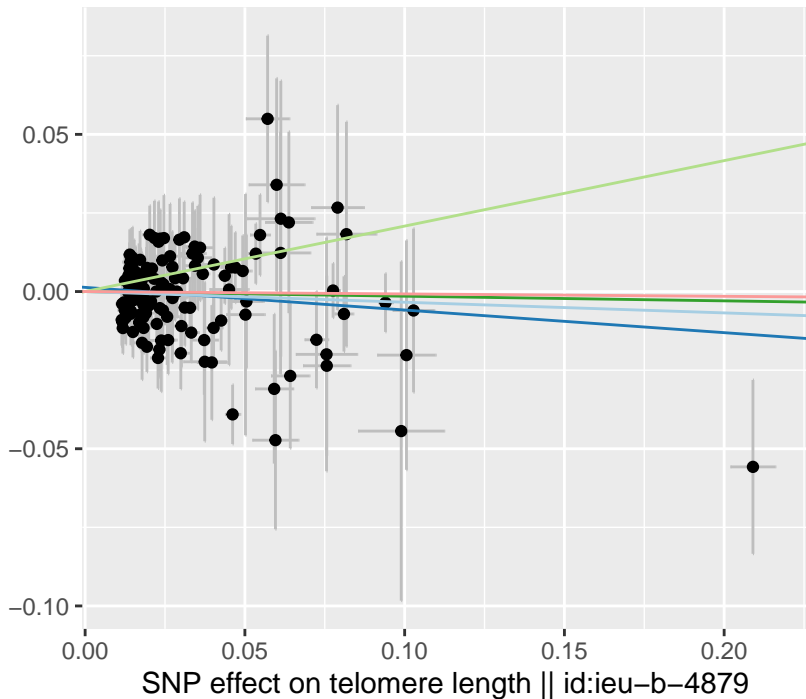

Supplement: Supplementary file 1 [file biomedicines-12-01874-s001.zip › Supplementary Figure/Figure S2/Scatter plot of MR analysis for TL to ubm-b-1553.pdf]

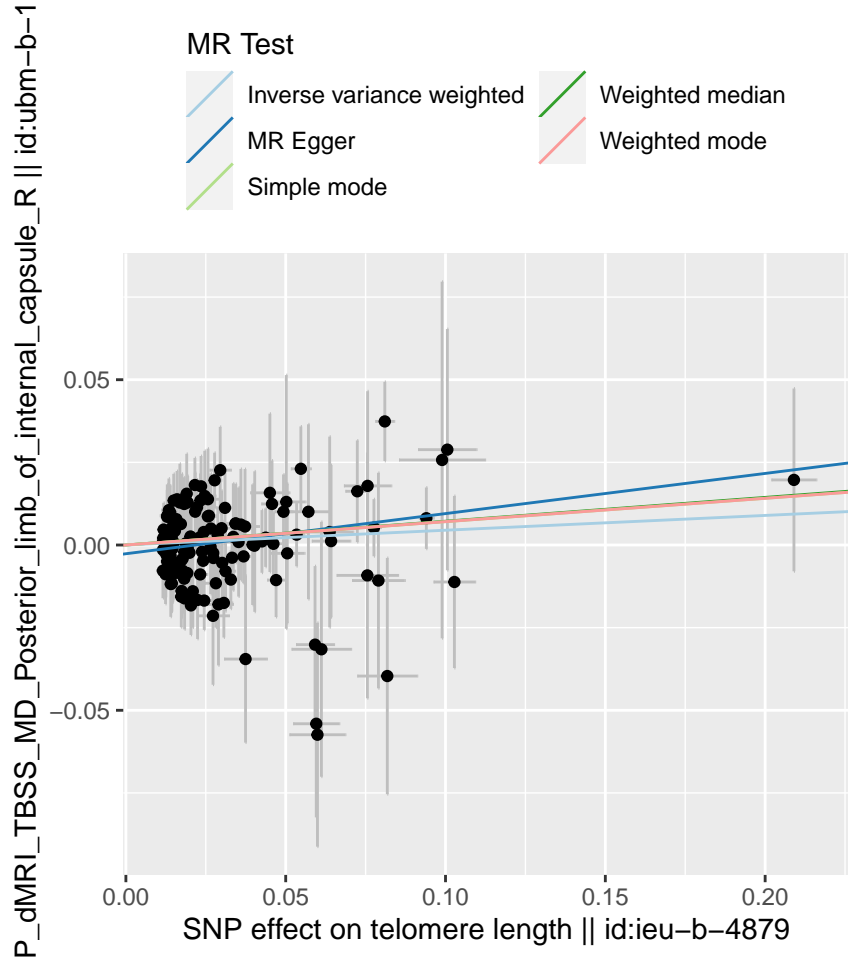

Supplement: Supplementary file 1 [file biomedicines-12-01874-s001.zip › Supplementary Figure/Figure S2/Scatter plot of MR analysis for TL to ubm-b-1620.pdf]

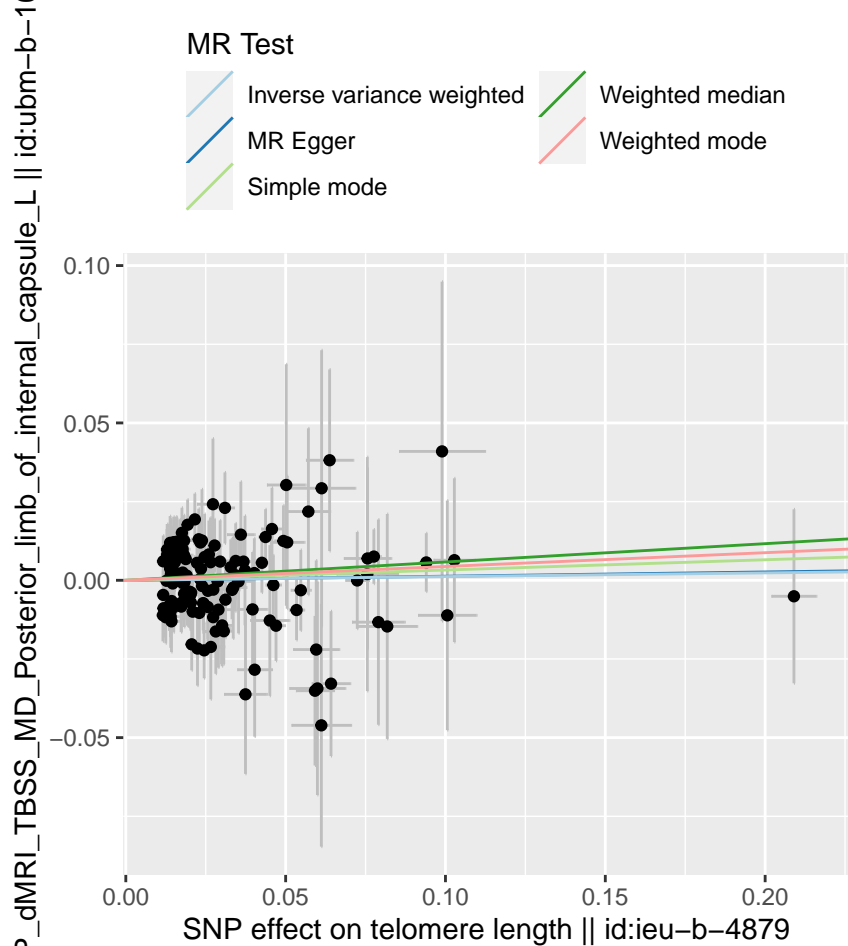

Supplement: Supplementary file 1 [file biomedicines-12-01874-s001.zip › Supplementary Figure/Figure S2/Scatter plot of MR analysis for TL to ubm-b-1621.pdf]

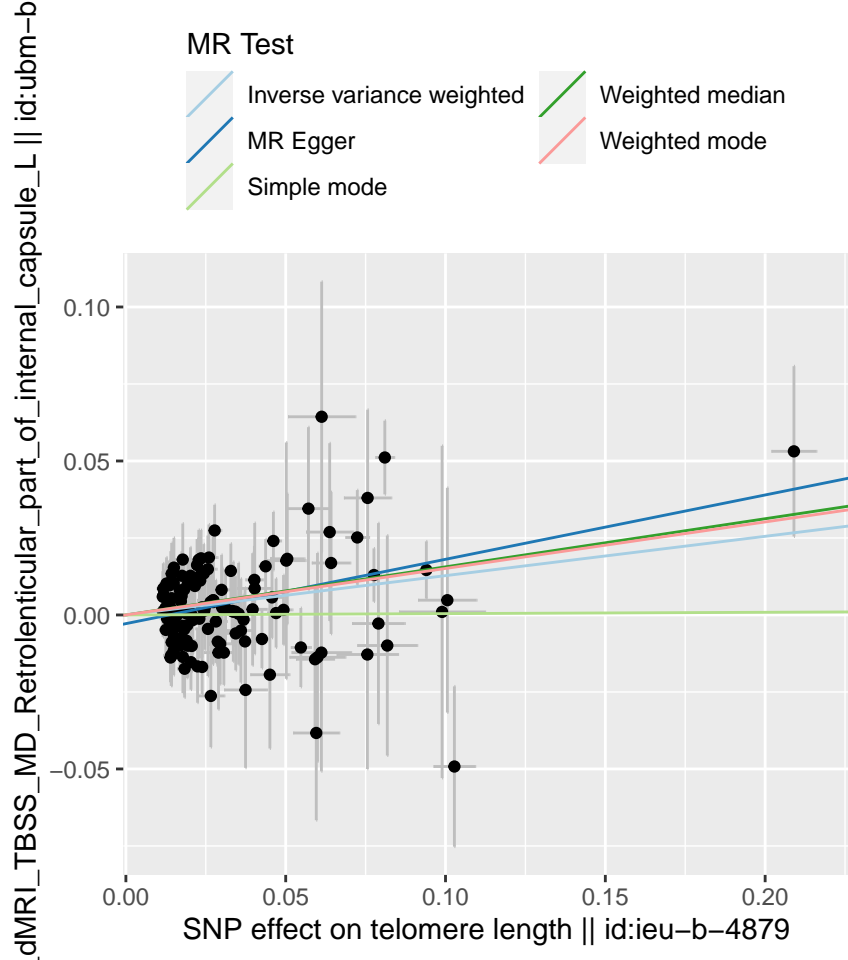

Supplement: Supplementary file 1 [file biomedicines-12-01874-s001.zip › Supplementary Figure/Figure S2/Scatter plot of MR analysis for TL to ubm-b-1623.pdf]

# MR Test

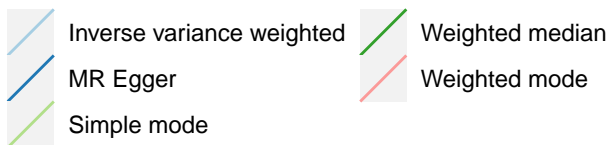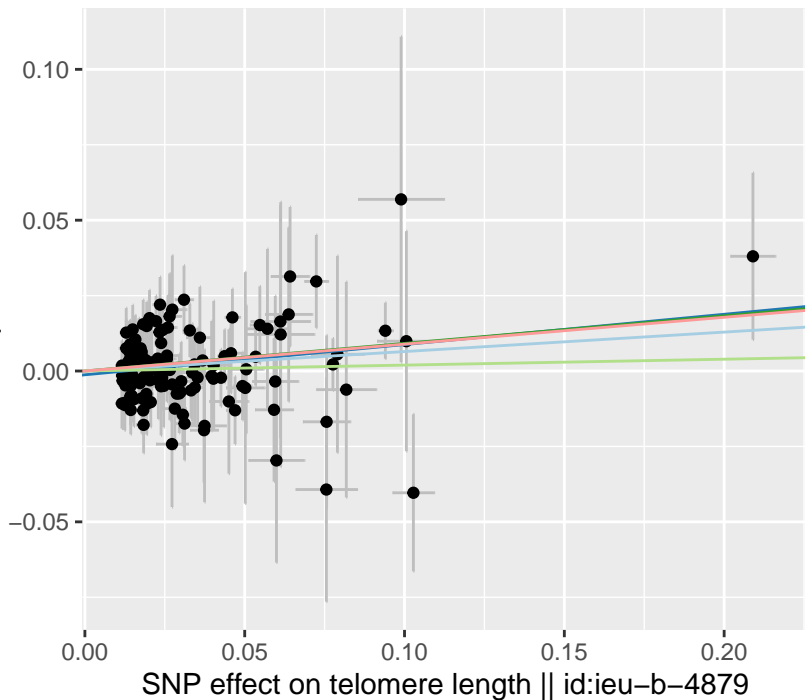

Supplement: Supplementary file 1 [file biomedicines-12-01874-s001.zip › Supplementary Figure/Figure S2/Scatter plot of MR analysis for TL to ubm-b-1626.pdf]

## MR Test

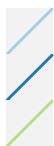

Inverse variance weighted

MR Egger

Simple mode

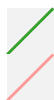

Weighted median

Weighted mode

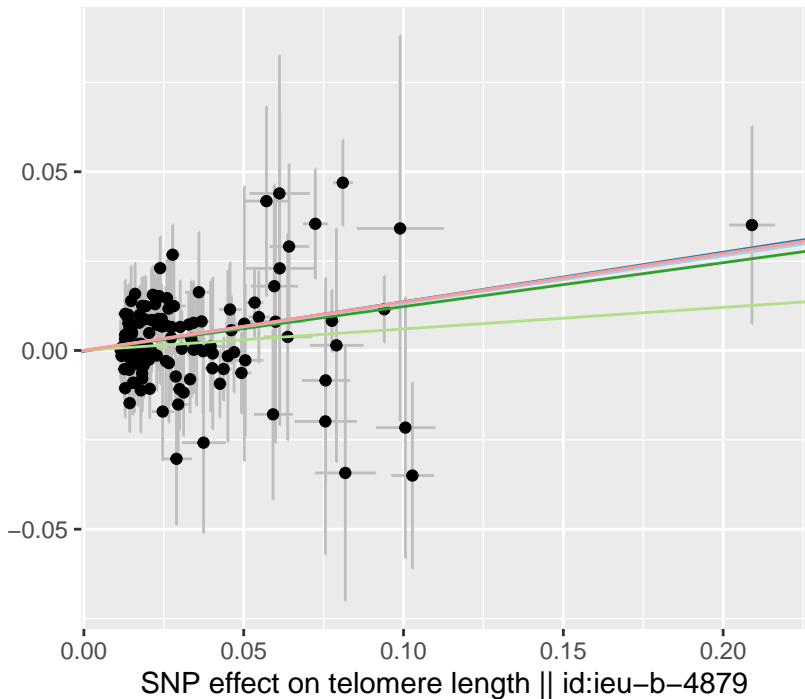

Supplement: Supplementary file 1 [file biomedicines-12-01874-s001.zip › Supplementary Figure/Figure S2/Scatter plot of MR analysis for TL to ubm-b-1628.pdf]
